# Two False Statements

**Published:** 1919-02-01

**Authors:** Arthur Stanley

**Affiliations:** Chairman, College of Nursing.


### THE EDITOR'S LETTER-BOX.

[Correspondence on all subjects is invited, but we cannot in any way be responsible for the opinions expressed by our correspondents, who must give their name and address as a guarantee of good faith, but not necessarily for publication. Correspondents are reminded that brevity of style and conciseness of statement greatly facilitate early insertion.]

### TWO FALSE STATEMENTS.

### Sir Arthur Stanley's Emphatic Denial.

*To the Editor of* The Hospital.

Sir,—My attention has been drawn to repeated statements made in the Press and elsewhere that the College of Nursing is placing members of the Voluntary Aid Detachments on its Register of Trained Nurses, and further that the Nation's Fund for Nurses, with which the College of Nursing is associated, is financing the Scholarship Scheme promoted by the Joint Committee of the British Red Cross Society and St. John of Jerusalem.

I take this opportunity once more of denying both these statements, emphatically. The College Register is formed entirely of nurses trained in General Nursing. The Nation's Fund is raised to endow the College, and to form a Benevolent Fund for nurses, whether members of the College or not, who may be in ill health, or who, from no fault of their own, stand in need of assistance.

I shall be obliged if you will give publicity to this letter.—I remain, Yours faithfully, Arthur Stanley, Chairman, College of Nursing.

